# Supplementary figures and images for: Functional Regrowth of Norepinephrine Axons in the Adult Mouse Brain Following Injury
Source: eNeuro. 2025 Jan 3;12(1):ENEURO.0418-24.2024. doi: 10.1523/ENEURO.0418-24.2024 (PMC11729145; doi:10.1523/ENEURO.0418-24.2024)

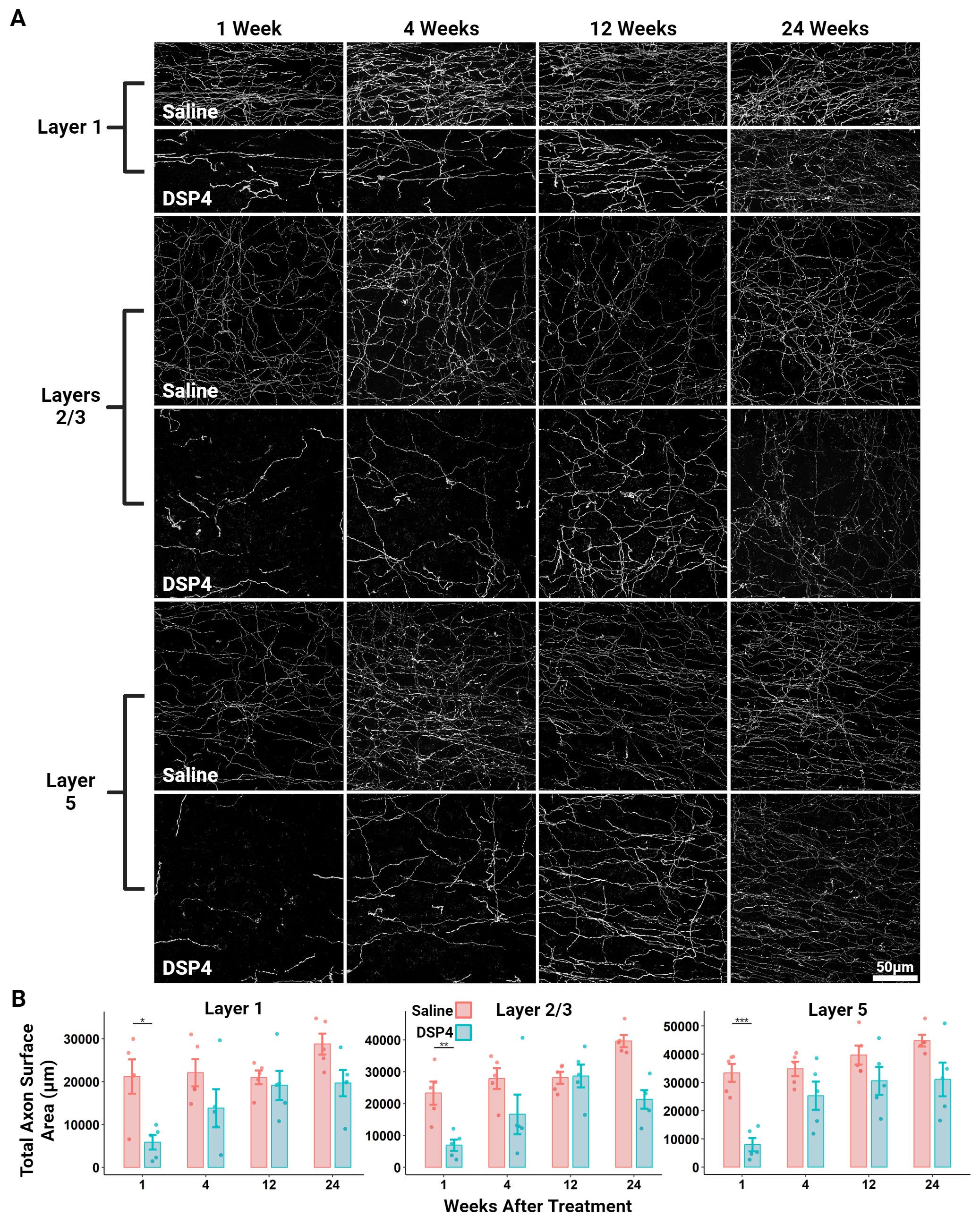

Supplement: Figure 1-1 — NE axons innervating the adult mouse primary somatosensory cortex regrow following a chemical lesion with the NE axon-specific neurotoxin DSP4. A, Representative 30µm maximum projected confocal stack images of layers 1, 2/3, and 5 of the primary somatosensory cortex in mice sacrificed 1-, 4-, 12-, and 24-weeks following treatment with DSP4 (50mg/kg) or saline. Dopamine-β-hydroxylase (DBH)-cre x mTmG mice were used to selectively label NE axons, neuronal tissue was sliced along the sagittal plane, and the native signal was amplified through processing with antibodies raised against GFP. B, IMARIS software was used to quantify the total axon surface area within 3-D reconstructed z-stacks (z=30µm) of layer 1, 2/3, and 5. Each plot symbol represents the total axon surface area of a single sagittal section of an individual mouse (n=5/group) and vertical bars show the standard error. * = P < 0.05; ** = P < 0.01; *** = P < 0.001. Download Figure 1-1, TIF file. [file eneuro-12-ENEURO.0418-24.2024-s001.tif]

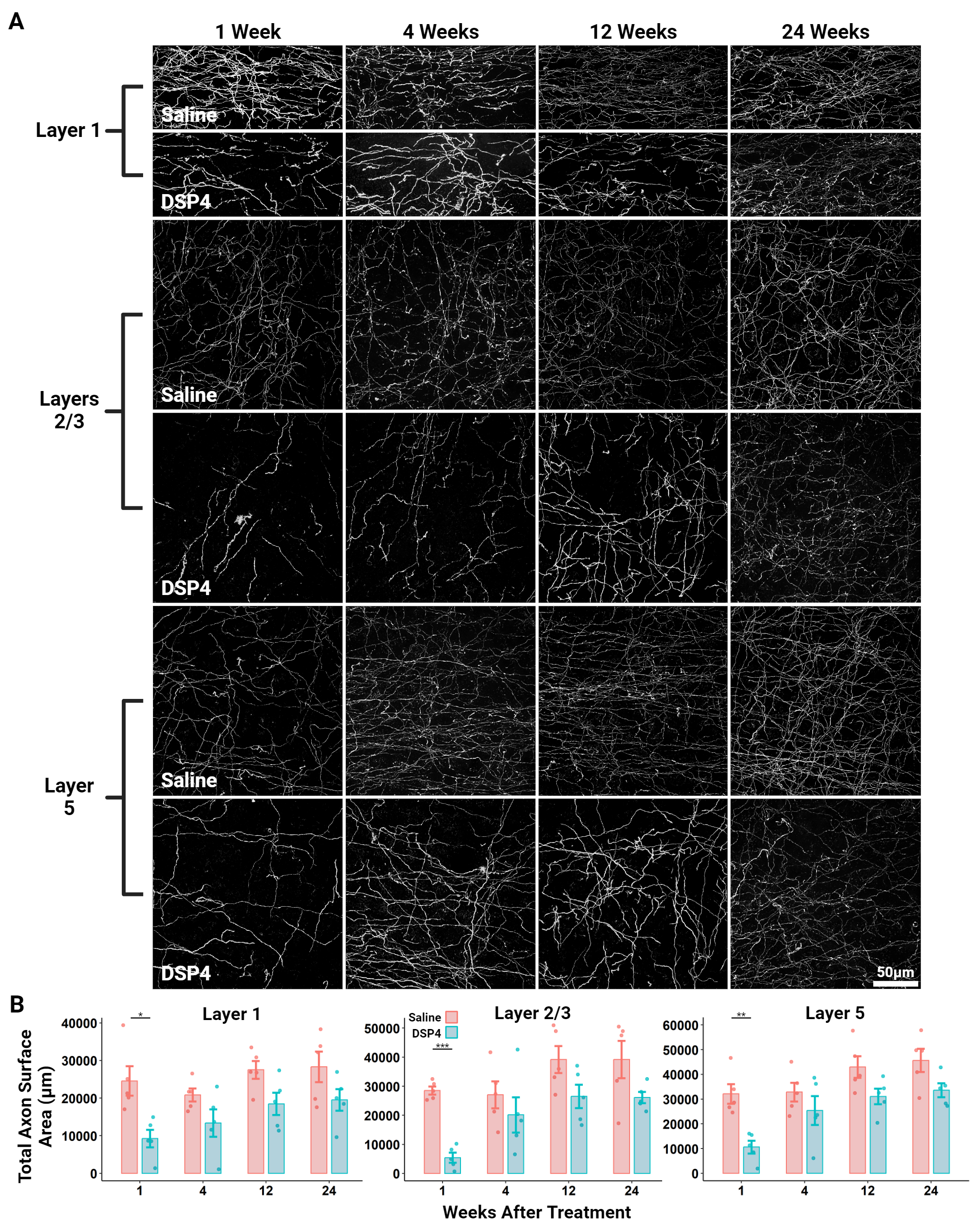

Supplement: Figure 1-2 — NE axons innervating the adult mouse primary motor cortex regrow following a chemical lesion with the NE axon-specific neurotoxin DSP4. A, Representative 30µm maximum projected confocal stack images of layers 1, 2/3, and 5 of the primary motor cortex of mice sacrificed 1-, 4-, 12-, and 24-weeks following treatment with DSP4 (50mg/kg) or saline. Dopamine-β-hydroxylase (DBH)-cre x mTmG mice were used to selectively label NE axons, neuronal tissue was sliced along the sagittal plane, and the native signal was amplified through processing with antibodies raised against GFP. B, IMARIS software was used to quantify the total axon surface area within 3-D reconstructed z-stacks (z=30µm) of layers 1, 2/3, and 5 of the primary motor cortex as demonstrated in A. Each plot symbol represents the total axon surface area of a single sagittal section of an individual mouse (n=5/group) and the vertical bars show the standard error. * = P < 0.05; ** = P < 0.01; *** = P < 0.001. Download Figure 1-2, TIF file. [file eneuro-12-ENEURO.0418-24.2024-s002.tif]

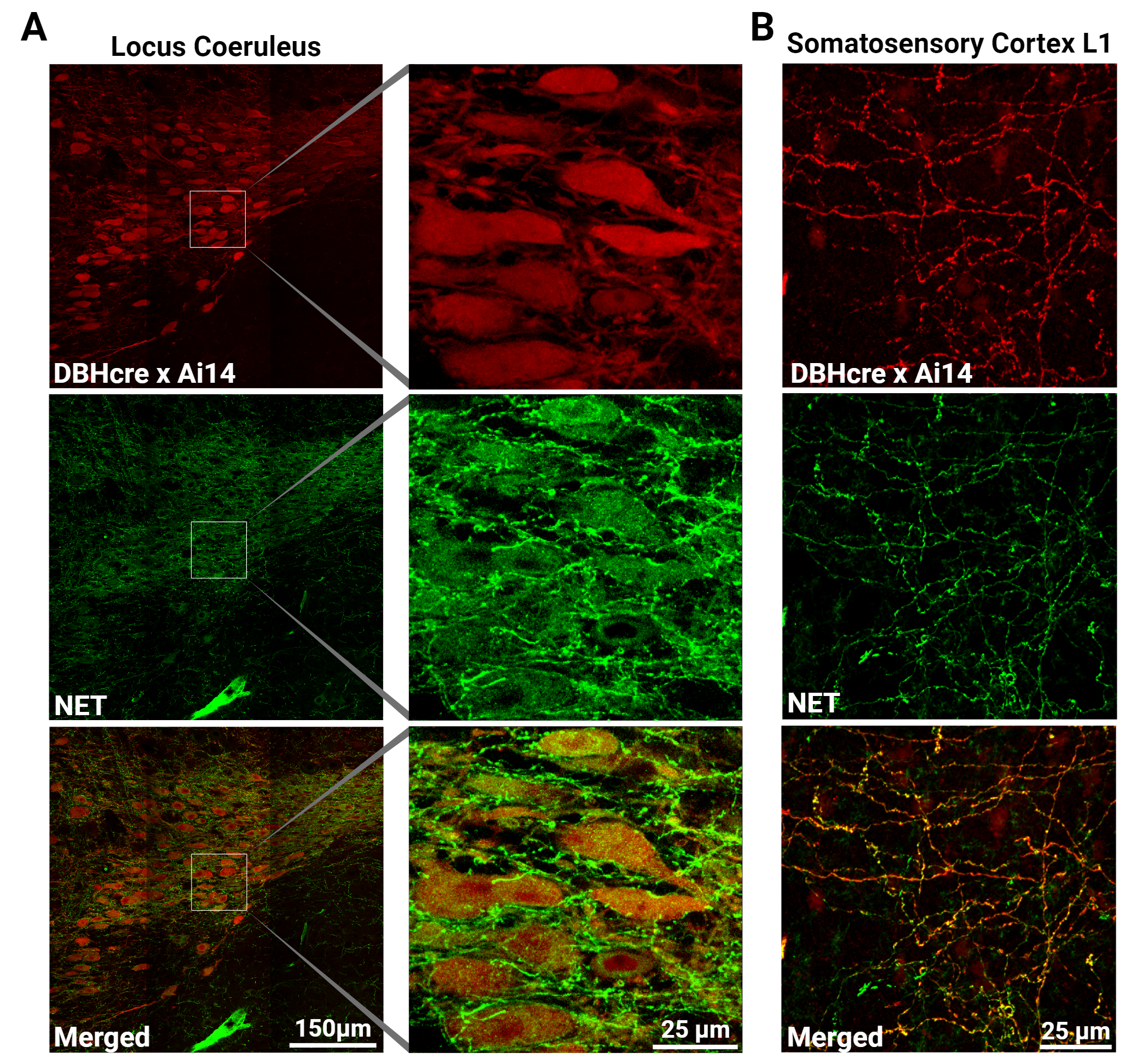

Supplement: Figure 2-1 — NE neurons are selectively labeled with TdTomato in dopamine-β-hydroxylase (DBH)-cre x Ai14 transgenic mice. A, Representative 3-by-3 tiled (left) and magnified subsection (right) 30µm maximum projected confocal stack images of the locus coeruleus. Brain tissue was sliced in the sagittal plane and counterstained with antibodies raised against the norepinephrine transporter (NET) to selectively label the plasma membrane of NE neurons. Ai14-driven tdTomato fluorescence was amplified with cross-reactive antibodies raised against DsRed. Ai14-positive cell bodies and axons consistently show NET immunoreactivity. B, Representative 30µm thick maximum projected confocal stack images of layer 1 of the primary somatosensory cortex section were processed in the same manner as those in panel A. Ai14-positive axons in this region consistently show NET immunoreactivity as well. Download Figure 2-1, TIF file. [file eneuro-12-ENEURO.0418-24.2024-s003.tif]

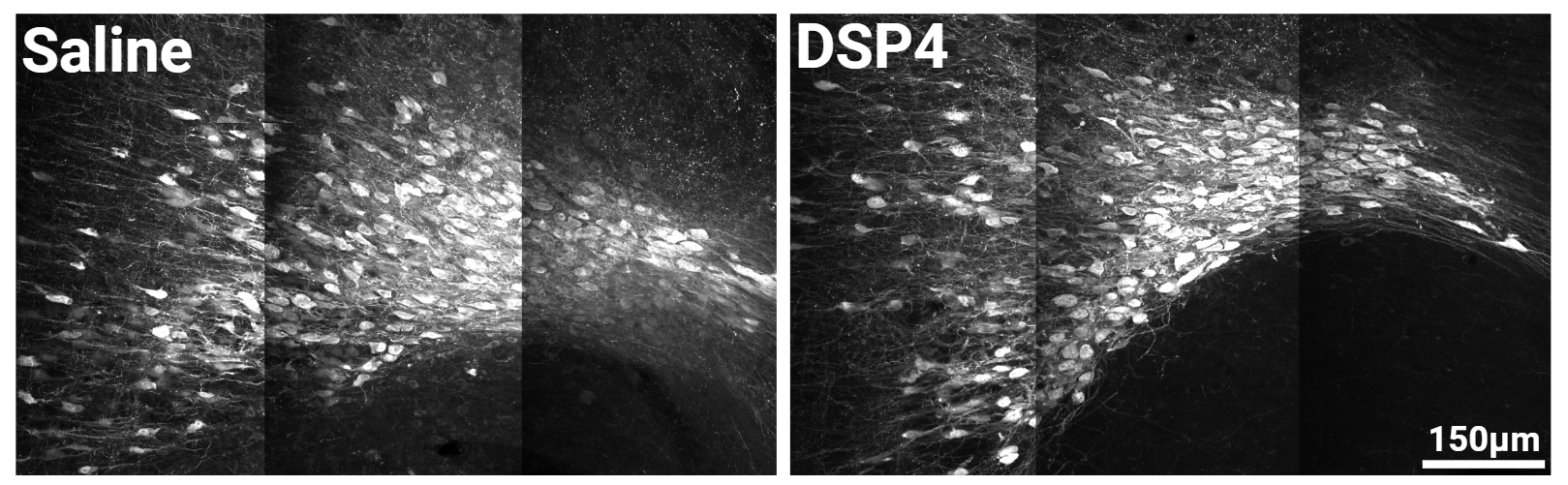

Supplement: Figure 2-2 — A single dose of DSP4 (50mg/kg), as used throughout the present study, does not lesion NE cell bodies within the locus coeruleus of the adult mouse. A, Representative 3-by-3 tiled 30µm maximum projected confocal stack images of the locus coeruleus 24-weeks following treatment with DSP4 (50mg/kg) or saline. DBHcre x Ai14 mice were used to selectively label NE neurons with tdTomato. The brain was sliced along the sagittal plane, and the tdTomato signal was amplified using cross-reactive antibodies raised against DsRed. IMARIS software was used to produce an exhaustive count of the Ai14-positive cell bodies within the locus coeruleus of individual animals. No significant difference in the number of cells was detected between the saline (1509 ± 204, n=8) and DSP4 (1246 ± 134 somata, n=9) groups (p = 0.289). The standard error is represented by vertical bars. Download Figure 2-2, TIF file. [file eneuro-12-ENEURO.0418-24.2024-s004.tif]

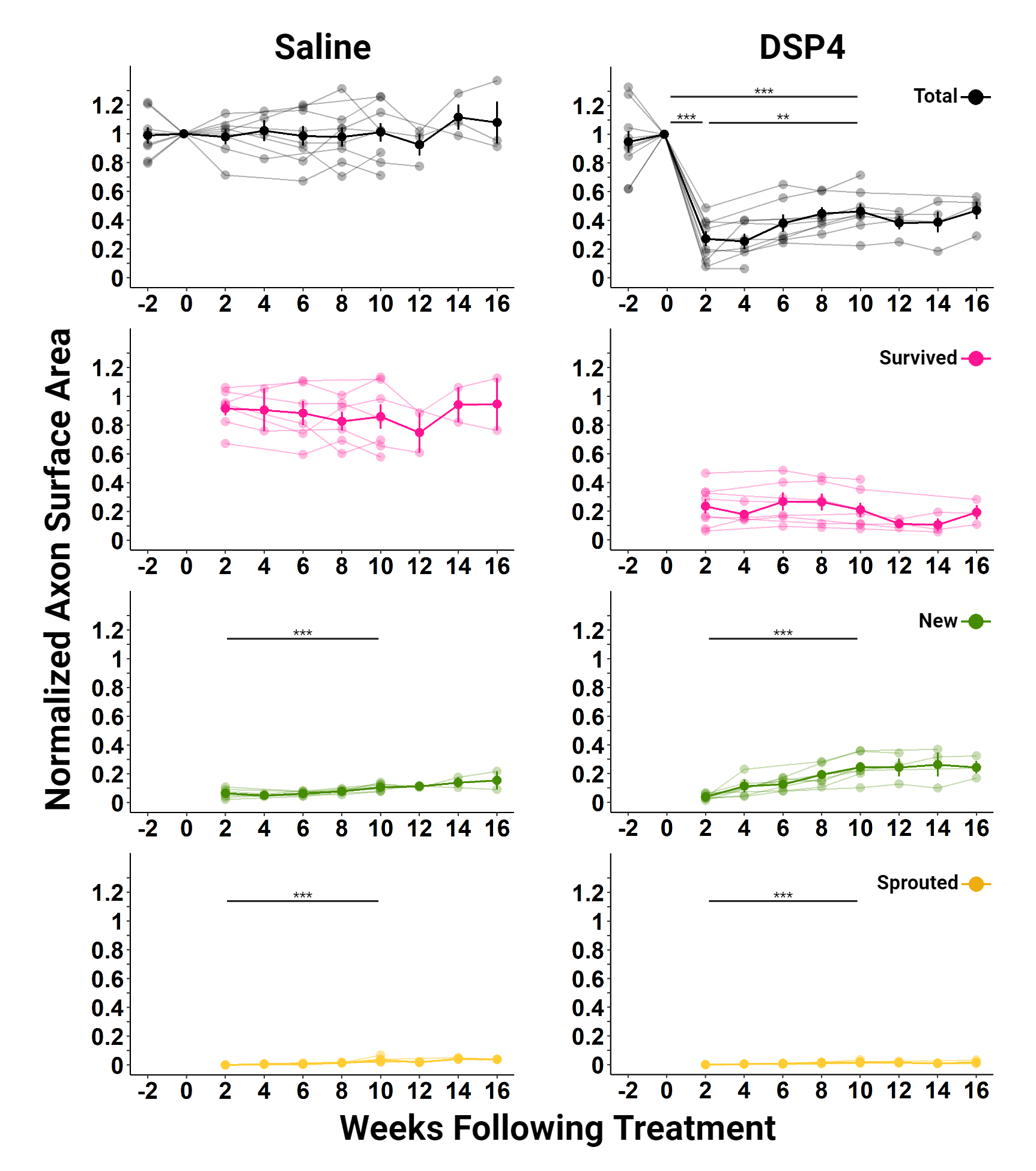

Supplement: Figure 2-3 — Long-term in vivo 2-photon imaging shows the regrowth of NE axons innervating the adult mouse somatosensory cortex following DSP4 treatment. Individual mouse (shaded) and population mean +/- standard error (solid) measurements of the axon surface area are shown. The population data are the same as illustrated in Figure 2B and are reproduced here to allow for comparison with the individual mouse measurements. Weeks during which data was able to be collected are indicated by shaded closed circles. Collection was sometimes limited by repair of the imaging rig and lines representing individual mice sometimes terminate due to deterioration of the optical quality of the imaging window. ** = P < 0.01; *** = P < 0.001. Download Figure 2-3, TIF file. [file eneuro-12-ENEURO.0418-24.2024-s005.tif]

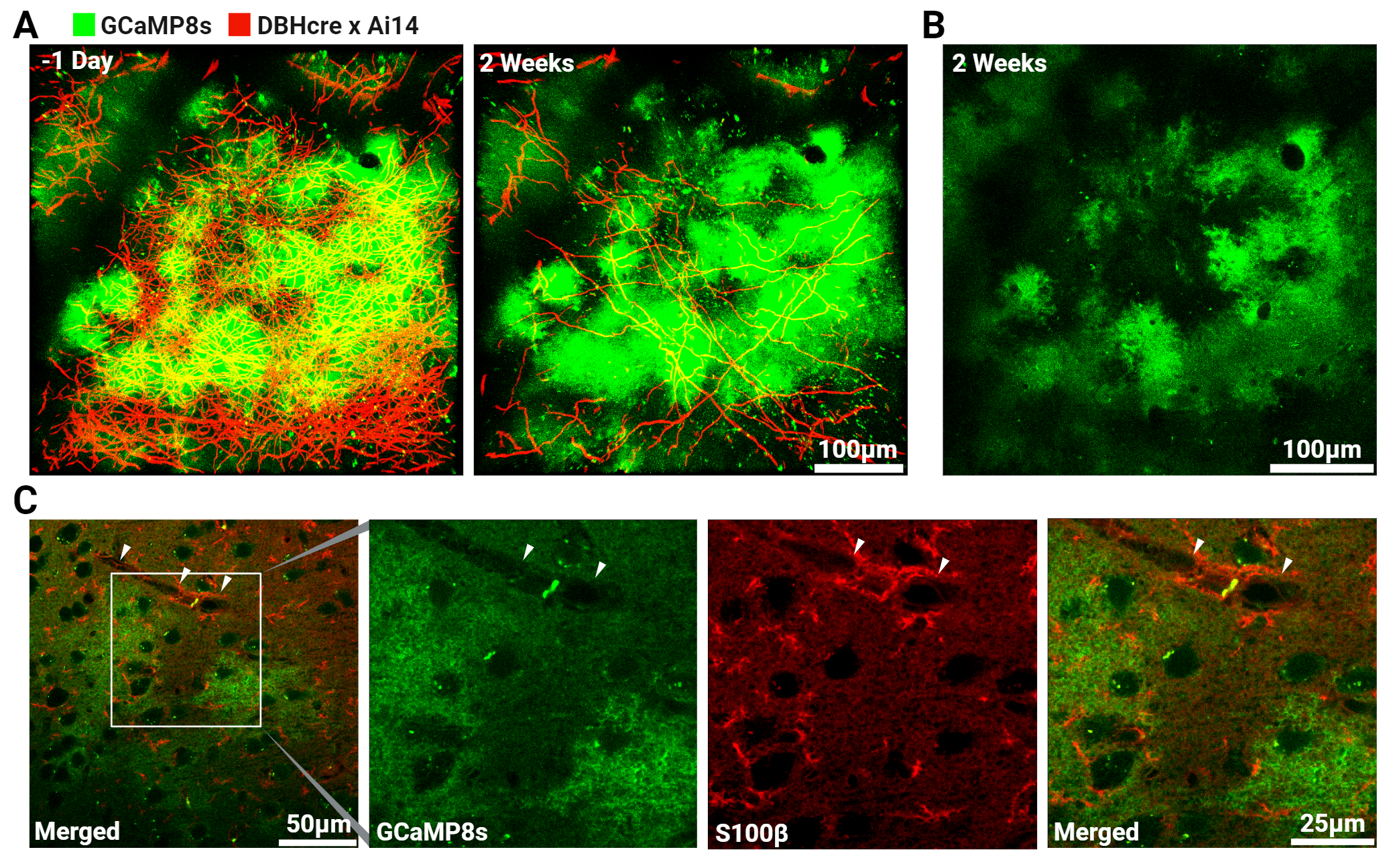

Supplement: Figure 4-1 — Infection with AAV5-gfaABC1D-jGCaMP8s induces expression of GCaMP8s in cortical astrocytes. A, Representative 108µm 3-D reconstructed images of the primary somatosensory cortex show virally induced GCaMP8s (green) and DBHcre x Ai14 (red) one day prior to and two weeks following lesion with the NE-specific neurotoxin DSP4. Viral injection 7 weeks prior to lesion induces patchy GCaMP expression which shows the tiled patterning characteristic of astrocytes. Note that the expression pattern and intensity of GCaMP was not significantly altered over the 15 day-long span encompassing the DSP4 challenge. B, 3µm-thick maximally projected z-stack images of the same GCaMP8s signal shown in A reveals the tendril-like processes characteristic of astrocytes. C, Representative single image of layer 2/3 of the primary somatosensory cortex 16 weeks following infection with AAV5-gfaABC1D-jGCaMP8s to selectively induce GCaMP8s in cortical astrocytes. Fixed brain tissue was sliced in the sagittal plane and processed with antibodies raised against the astrocyte marker S100β. As in A and B, the GCaMP8s signal demonstrates a patchy transfection pattern and tiling while S100β labels all astrocytes, highlighting particular cytoskeletal elements. White arrowheads indicate a capillary contacted by S100β-positive astrocyte endfeet. Download Figure 4-1, TIF file. [file eneuro-12-ENEURO.0418-24.2024-s006.tif]
